# Immediate Effect of Masticatory Muscle Activity with Transcutaneous Electrical Nerve Stimulation in Muscle Pain of Temporomandibular Disorders Patients

**DOI:** 10.3390/jcm9103330

**Published:** 2020-10-16

**Authors:** Susumu Abe, Akane Miyagi, Kaoru Yoshinaga, Yoshizo Matsuka, Fumihiro Matsumoto, Emi Uyama, Yoshitaka Suzuki, Masamitsu Oshima, Kazuo Okura, Eiji Tanaka

**Affiliations:** 1 Department of Comprehensive Dentistry, Tokushima University Graduate School of Biomedical Sciences, Tokushima 7708504, Japan; minkichi@mc.pikara.ne.jp; 2 Department of Orthodontics and Dentofacial Orthopedics, Tokushima University Graduate School of Biomedical Sciences, Tokushima 7708504, Japan; rururu1214@outlook.com (K.Y.); etanaka@tokushima-u.ac.jp (E.T.); 3 Department of Stomatognathic Function and Occlusal Reconstruction, Tokushima University Graduate School of Biomedical Sciences, Tokushima 7708504, Japan; matsuka@tokushima-u.ac.jp (Y.M.); yosuzuki@tokushima-u.ac.jp (Y.S.); m-oshima@tokushima-u.ac.jp (M.O.); okura.kazuo@tokushima-u.ac.jp (K.O.); 4 Department of Oral Medicine, Institute of Biomedical Sciences, Tokushima University Graduate School, Tokushima 7708504, Japan; fumihiro@tokushima-u.ac.jp; 5 Department of Biomaterials and Bioengineering, Tokushima University Graduate School of Biomedical Sciences, Tokushima 7708504, Japan; uyamanikofu@tokushima-u.ac.jp

**Keywords:** transcutaneous electrical nerve stimulation, muscle pain, temporomandibular disorders

## Abstract

Transcutaneous electrical nerve stimulation (TENS) is a non-invasive treatment modality for acute and chronic pain. However, little information for muscle activity is available on the immediate effects of TENS in masticatory muscle pain related to temporomandibular disorders (TMDs). The present study aimed to evaluate the immediate effects of TENS treatment on TMD-related muscle pain. Thirty-six patients with TMD-related muscle pain and 39 healthy subjects served as TMD and control groups, respectively. For objective evaluations, maximum mouth opening, and maximum bite force were measured before and after TENS. The pain intensity was assessed according to a 100-mm visual analog scale (VAS). TENS was applied to painful muscles for 20 min with frequencies of 100–200 Hz. The treatment outcome was evaluated using Global Rating of Change (GRC) scales. In the TMD group, VAS values significantly decreased after TENS. Although there was significant increase in the maximum mouth opening after TENS for only TMD group, the maximum bite force of both groups was significantly greater after TENS. According to GRC scales, one patient with TMD-related muscle pain expressed negative feelings after TENS. Conclusively, TENS treatment might quickly relieve pain in masticatory muscles and improve masticatory functions in patients with TMD-related muscle pain.

## 1. Introduction

In the field of clinical dentistry, temporomandibular disorders (TMDs) is one of the major diseases. TMDs have been characterized as chronic orofacial pain conditions affecting around 20% to 25% of the population, and one-fifth of those with TMD-related pain and dysfunction require treatment [1,2].

The most common symptom of arthritic temporomandibular joint conditions is musculoskeletal pain, which is characterized as articular or muscular pain. In epidemiological surveys, TMD-related facial pain has been found to occur in 4–12% of the population [3,4,5,6], and severe symptoms are reported by 10% of the subjects [7]. In a large study population, tenderness of masticatory muscles and temporomandibular joints was found in 15% and 5%, respectively [8], but these symptoms are self-reported by about 4% and 6–8%, respectively [9,10]. These results suggest that patients might not have so bothersome muscular tenderness but muscular clinical symptoms potentially. This masticatory muscle tenderness or muscle pain is characterized by a long-lasting dull ache or fatigue localized in the jaw and temporal muscles due to muscular overload, e.g., by daytime teeth-clenching or sleep bruxism [11,12]. Furthermore, masticatory muscle pain may arise from protective reflex contractions involving the soft tissue around the affected joint [13]. Since masticatory muscle pain and masticatory dysfunction affect the oral health-related quality of life [14], dental clinicians should remove TMD pain quickly from the patients, resulting in an increase of masticatory function, because long duration of pain causes reduction of quality of life [15].

In TMD patients with pain, it is indispensable to make an accurate diagnosis and reduce or eliminate the pain in the first place. Dental clinicians should treat these patients by selecting the adequate therapy type for pain relief, such as patient education, pharmacotherapy, and physiotherapy [14,15]. Often, pain management does not only consist of a single therapy but may involve combination therapies. Physical therapy modalities can be selected in combination with other therapies to reduce pain. Superficial moist heat or localized cryotherapy may relieve pain sufficiently to permit therapeutic exercises which are designed to increase muscle strength, reduce joint contractures, and maintain a functional range of motion. Ultrasound, electrogalvanic stimulation, and massage techniques are also helpful in reducing pain [16]. However, no physical therapy induces a fast reduction of TMD-related masticatory muscle pain.

Transcutaneous electrical nerve stimulation (TENS) uses an electrical device to stimulate the nerves and is one of the safest and most reasonable modalities that can reduce both chronic and acute pain [17]. TENS-induced pain inhibition is a known application of the gate control theory [18]. This theory explains that the activation of the pain conducted in thin fibers (Aδ- and C-fibers) were inhibited though the medium of the transmission cells according to raise activation in inhibitory interneuron, which Aβ afferent fibers in the posterior horn of the spinal cord are activated [19]. The electrical stimulation inhibits the transmission of painful impulses in the spinal cord and stimulates the release of endogenous opioids in the brain [20]. This strategy of TENS treatment has already been successfully applied to reduce non-odontogenic orofacial pain [21,22,23,24]. Additionally, a previous study reported TENS treatment on TMD patients had improved the electromyograph (EMG) activity of chewing in comparison with TENS placebo TMD patients [25]. Another previous study had reported TENS treatment had muscular relaxation effect for TMD and healthy control subjects using EMG [24]. In short, it might be possible for TENS treatment to affect the muscle activity as well as the pain reduction. However, little information is available on a quick effect of TENS in masticatory muscle pain and activity involved in TMDs.

Thus, we hypothesized that TENS quickly attenuates masticatory muscle pain related to TMDs and enhances stomatognathic function including bite force and mouth opening. The present study aimed to evaluate the immediate effects of TENS treatment in TMD patients with masticatory muscle pain and whether TENS treatment affected masseter muscle activity. The clinical implications through this finding will be discussed for TENS treatment in TMD muscle pain.

## 2. Experimental Section

### 2.1. Participants

Thirty-six patients with TMD-related muscle pain (TMD group), who had been referred to the Temporomandibular Joint Clinic, Tokushima University Hospital, were recruited to this study from October 2018 to July 2020. The inclusion criteria for enrollment were age over 18 years, a diagnosis of muscle pain according to the diagnostic criteria for TMD (DC/TMD) [26], and pain lasting for more than 1 month. Ten muscles were palpated according to the DC/TMD, including the deep and superficial masseter, the anterior and posterior parts of the temporalis muscle, the posterior belly of the digastric muscle, the sternocleidomastoid muscle, and the trapezius. Especially, the masseter and temporalis muscles were carefully assessed using a Palpeter (SUNSTAR Co., Ltd., Osaka, Japan) [27]. The exclusion criteria comprised contraindications for electrical stimulation, systemic muscular or arthritic diseases, neuropathic pain or neurological disorders, pain of dental origin, and use of non-steroidal anti-inflammatory drugs as analgesics. Thirty-nine healthy individuals were enrolled as control group. They were recruited to the study by screening healthy patients who attended regular dental checkups at Tokushima University Hospital or staff persons at this Hospital. They did not have a history of TMD and did not have TMD-related muscle pain during the study period. Similar to the TMD group, healthy subjects were examined by the same methods using the Palpeter and adhered to the same exclusion criteria as TMD patients.

The Ethics Committee of Tokushima University Hospital approved the study, and written informed consent from each participant was obtained after a full explanation of the research purposes and procedures (No. 3241).

### 2.2. Pretreatment and Posttreatment Examinations

Dental clinicians who had enough training to manage TMD determined the present pain intensity in a medical interview. The pain intensity of each patient was assessed using a 100-mm visual analog scale (VAS). The VAS measures the painful experience using a straight line of 100 mm, with the left margin anchored by the term “no pain” and the right by the term “worst imaginable pain” [28].

For the objective evaluation before and after TENS treatment, the degree of mouth opening was determined in a state without activity-induced muscle pain between the upper and lower incisors using a measuring scale. Furthermore, the bite force was measured unilaterally on the affected side in the first molar region using a portable occlusal force gauge (occlusal force meter GM10; Nagano Keiki Co., Ltd., Tokyo, Japan). In the control group, the bite force was assessed on the right side. The subjects were seated with the Frankfort plane approximately parallel to the floor and instructed to bite on the biting element as hard as possible for 3 s. The force gauge displayed the highest recorded value. The bite force was measured twice with a 30 s interval between the attempts, and the mean value of the two measurements was used as the representative value.

### 2.3. TENS Treatment

**Figure 1:**
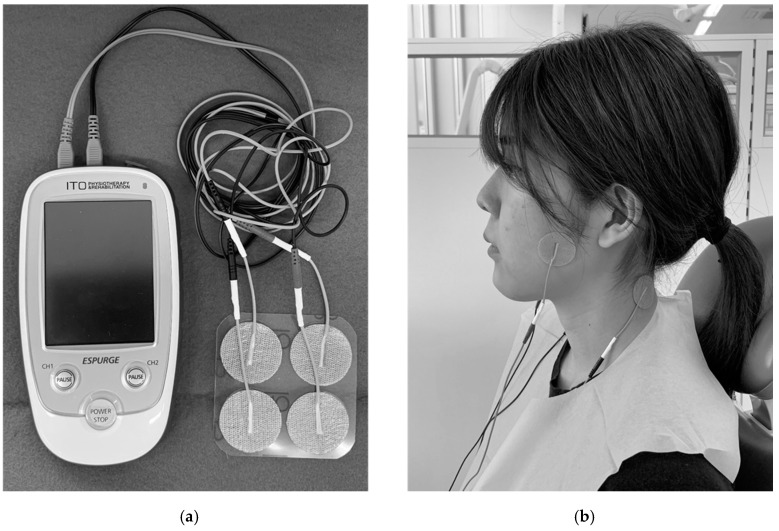
Clinical trial of transcutaneous electrical nerve stimulation (TENS) for pain relief in masticatory muscles. (**a**) The TENS device ESPURGE (ITO CO., LTD, Tokyo, Japan) is used because it is a portable device and easy to use at the chairside. (**b**) Four electrodes are applied to the skin for 20 min at a phase duration of 50 μs with frequencies of 100–200 Hz (sweep modulation mode).

After the pretreatment examination, the patients received the TENS treatment by dental clinicians. The patients sat in a dental chair in a quiet room during this intervention. Using a dental TENS device (ESPURGE; ITO Co., Ltd., Tokyo, Japan; Figure 1a), the current was applied via electrodes to the skin for 20 min [29] at a phase duration of 50 μs, and the frequency settings were in the sweep modulation mode ranging from 100–200 Hz. The applied waveform was a biphasic rectangular pulse, and the output current was above the sensory threshold. Two electrodes were placed on the masseter muscles exhibiting the pain and the back of the neck (Figure 1b). The dental clinicians selected the output power (intensity) of the device based on the patients’ sensitivity and tolerance thresholds so that the stimulation was not painful and without any side-effects such as twitching of the eyelids.

After the TENS treatment, the enrolled subjects were examined using the Global Rating of Change (GRC) scale, in which the patients marked an 11-point scale on a self-completed questionnaire in order to evaluate their total clinical changes [30]. The subjective evaluation ranged from “Very much worse” over “Unchanged” to “Very much improved” with corresponding numbers ranging from −5 to +5.

Immediately after the TENS treatment, the pain intensity was re-evaluated using the VAS. In addition, the maximum mouth opening and bite force were measured again using the same procedure as in the pretreatment examination.

### 2.4. Statistical Analysis

Data were analyzed using SPSS 22.0 (SPSS Inc., Chicago, IL, USA). Their normality was assessed by the Shapiro-Wilk test and an evaluation of the box plot. If normality of the data distribution was confirmed, data were expressed as the mean and standard deviation (SD). Otherwise, data were expressed as the median and interquartile range (IQR: first quartile–third quartile). The difference of the sex ratio between the two groups was evaluated using Pearson’s chi-squared test. The Wilcoxon signed rank sum test was used to compare the VAS values before and after TENS application only in the TMD group because the control group did not experience muscle pain. For the values of maximum mouth opening and bite force before and after TENS application, general linear model (GLM) analyses were carried out for both the control and TMD groups. If there was significant interaction, the paired t-test was performed to compare before and after TENS application as post-hoc test. Furthermore, GRC scales data were assessed using the Mann-Whitney U test, because they were ordinal data. Probabilities below 0.05 were considered statistically significant.

Since some previous reports were various sample size, the present study estimated the calculated necessary estimates for sample size. The effect size was used for the convenient statistical type of parametric or non-parametric test. The effect size of comparison between two groups was considered as small (0.20), medium (0.50), and large (0.80) [31]. The statistical power (1-β) was calculated using the G*Power software. The total number of estimated sample size was calculated from 52 to 152 subjects using medium and large of prospective effect size. A power analysis was carried out to calculate after statistical analysis as post-hoc test. The partial square ETA of GLM was used as a measure of the effect size in this study. The power was presented with three decimal places.

## 3. Results

### 3.1. Participants

The TMD group consisted of 9 males and 27 females ranging in age from 19 to 82 years (mean ± SD, 56.8 ± 17.2 years). The control group included 17 males and 22 females ranging from 24 to 84 years (52.5 ± 17.6 years). The sex ratio was also not significantly different between the two groups (*p* = 0.09). There was no significant difference in age between the TMD and control groups (*p* = 0.82). For male participants, there was no significant difference in age between the TMD and control groups (51.3 ± 25.3 years and 55.3 ± 15.7 years, respectively; *p* = 0.67). Similarly, for female participants, no significant age difference was detected between the two groups (58.6 ± 13.7 years in the TMD group and 50.3 ± 19.0 years in the control group; *p* = 0.09) (Table 1).

Because the output power of this device was adjusted to suit each participant based on their sensitivity, there were no significant differences in power between TMD patients and control subjects (*p* = 0.28, Table 1).

### 3.2. Pain Intensity

**Figure 2:**
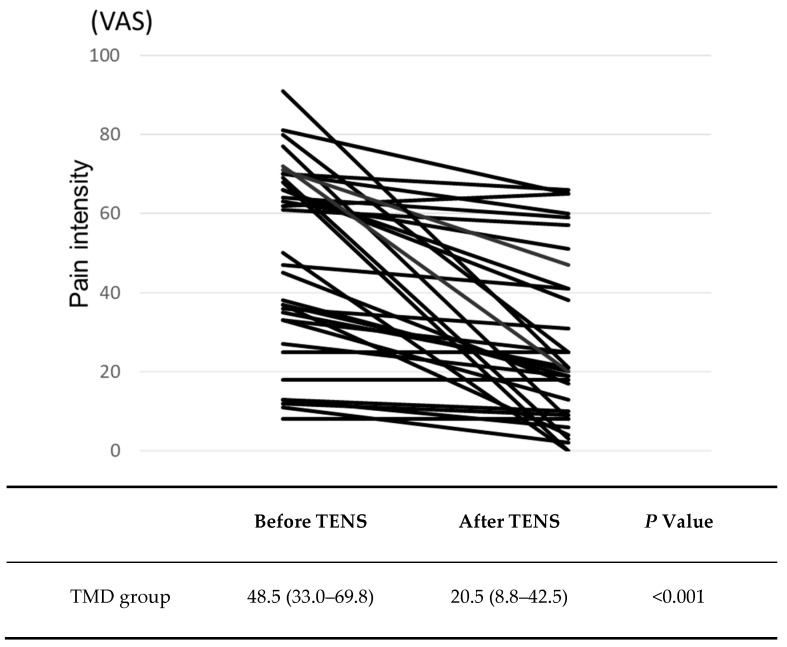
Changes in pain intensity before and after transcutaneous electrical nerve stimulation (TENS) treatment. Data are the Median (IQR: first quartile–third quartile). There was a significant decrease in the mean visual analog scale (VAS) value after the TENS treatment (Wilcoxon signed rank sum test).

For the patients with TMD-related muscle pain, the VAS value for pain intensity before the TENS treatment was 48.5 (33.0–69.8), ranging from 8.0 to 91.0, and there was significant (*p* = 0.001) decrease in the VAS values after the TENS treatment (20.5 (8.8–42.5), ranging from 0.0 to 66.0; Figure 2). The average pain intensity was reduced by 41.1%. Three patients had the same VAS values before and after the TENS treatment. In one patient, the VAS value after TENS treatment was increased in comparison to that before TENS treatment. The effect size of this result was large, and Power was 0.995.

### 3.3. Objective Evaluation

#### 3.3.1. Maximum Mouth Opening without Pain

**Figure 3:**
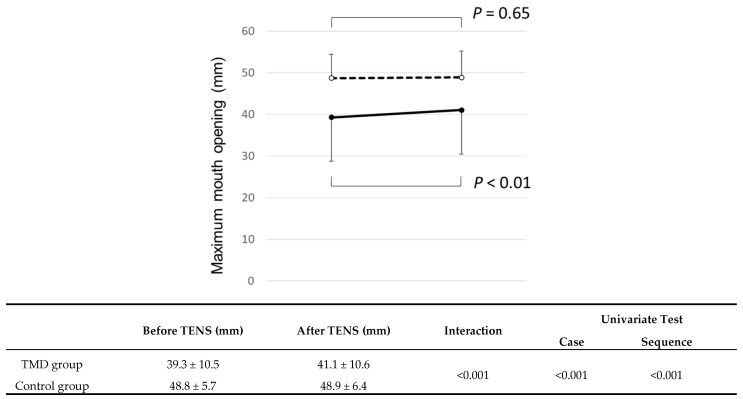
Maximum mouth opening before and after transcutaneous electrical nerve stimulation (TENS) application. Data are the mean ± standard deviation. The solid line indicates the temporomandibular disorder (TMD) group, and the dotted line indicates the control group. Case indicates the comparison between TMD and control groups, whereas sequence means the comparison between before and after treatment. There was no significant difference in maximum mouth opening between the two groups (General linear model analysis).

There was significant interaction between the two groups (*p* < 0.001), indicating that both TMD and control groups had the same trend in the change before and after TENS application (Figure 3). Since there was significant interaction between two groups, the comparison between before and after TENS intervention was carried out for each group as a post-hoc test. For the TMD group, the maximum mouth opening without pain was 39.3 ± 10.5 mm before the TENS treatment, and there was significant increase in this parameter after TENS treatment (41.1 ± 10.6 mm, *p* < 0.001). The maximum mouth opening without pain was increased by 7.5% for the TMD group. However, for the control group, the values for maximum mouth opening were 47.9 ± 10.6 mm and 48.2 ± 6.9 mm before and after TENS intervention, respectively. These values were not significantly different (*p* = 0.65). The partial square ETA of interaction was large, and Power was 0.977. Furthermore, the effect size in TMD patients was large, and Power was 0.883.

#### 3.3.2. Maximum Bite Force

**Figure 4:**
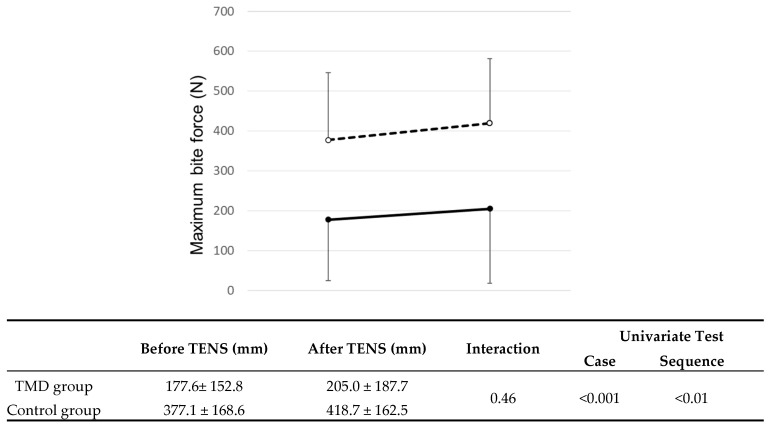
Maximum bite forces before and after transcutaneous electrical nerve stimulation (TENS) application. Data are the mean ± standard deviation. The solid line indicates the temporomandibular disorder (TMD) group, and the dotted line indicates the control group. There were significant differences in maximum bite force between TMD and control groups (*p* < 0.001) and between before and after TENS application (*p* < 0.01) as a univariate test (General linear model analysis).

In the TMD group, the maximum bite force was 177.6 ± 152.8 N before the TENS treatment, and was increased to 205.0 ± 187.7 N after TENS treatment. In the control group, the values for the maximum bite force before and after TENS intervention were 377.1 ± 168.6 N and 418.7 ± 162.5 N, respectively. The maximum bite fore was increased by 13% and 16% for the TMD group and the control group, respectively. There was no significant interaction between the two groups (*p* = 0.46), indicating that both TMD and control groups had the same trend in the change of the maximum bite force before and after TENS application. However, both the case and the sequence had statistically significant differences (*p* < 0.001 and *p* <0.01, respectively, Figure 4), meaning that significant differences of the maximum bite force were found between TMD and control groups for the case, as well as between before and after TENS treatment for the sequence.

### 3.4. GRC Scales

**Figure 5:**
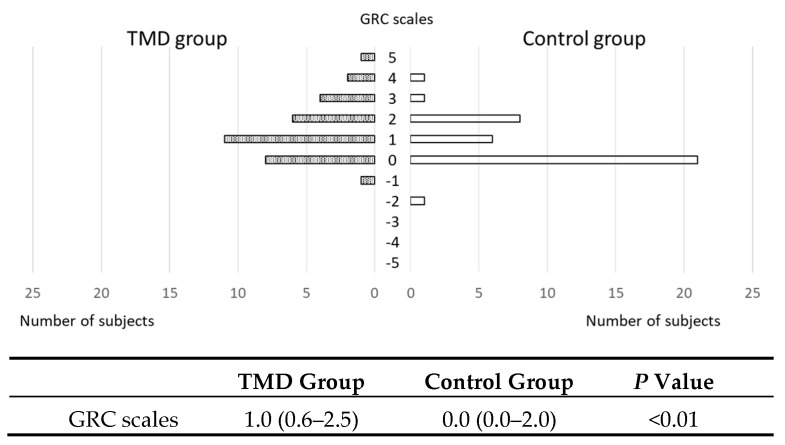
Distribution of the scores in the Global Rating of Change (GRC) scales for temporomandibular disorder (TMD) and control groups. Shaded bars indicate the numbers of subjects for each GRC scales score in the TMD group, and white bars represent those in the control group. Data are presented as the median value (IQR: first quartile–third quartile). There was a significant difference in GRC scales scores between TMD and control groups (*p* < 0.01; Mann-Whitney U test).

One female patient with TMD-related muscle pain expressed negative feelings (score of −1) after TENS treatment, whereas one female of the control group had the impression that she got a little worse (score of −2). Both subjects were due to itching which was aggravated after TENS intervention. Except for these participants, the TMD and control group showed scores of 0 or higher in the GRC scales, meaning that the responses ranged from “unchanged” to “Very much improved”. There was a significant difference in GRC scales scores between TMD and control groups (*p* < 0.01; Figure 5). Because the standardized test statistic (Z score) from Mann-Whitney U test was low and the assuming effect size was middle, however, power value was 0.28.

## 4. Discussion

In the present study, we found that TMD patients subjectively reduced masticatory muscle pain by TENS treatment and slightly changed good clinical efficacy using GRC scales in comparison with before TENS treatment. Furthermore, stomatognathic function including maximum mouth opening without pain and maximum bite force quickly improved after TENS treatment in TMD patients. These findings suggest that the TENS not only removes masticatory muscle pain but also improves masticatory function.

The present study employed TMD patients with masticatory muscle pain diagnosed by DC/TMD and gender- and age-matched healthy control subjects. Furthermore, their intervened output power of device was almost the same between the TMD and control groups. Thus, both TMD and control groups were considered as the same conditions.

The quick effects of TENS treatment on masticatory muscle pain related to TMDs could be associated with the alternate frequency protocol adopted in the present study. TENS might be effective as an adjunct treatment for masticatory muscle pain, especially, because it is inexpensive and has a favorable safety profile compared with long-term medication. In the present study, more than 88% of the patients with TMD-related muscle pain experienced a reduction in pain sufficient to allow maximum clenching, although the remaining less than 12% reported no changes or increase in pain after TENS. In previous studies for muscle pain treatment with TENS, therapeutic efficacy of TENS was shown by self-reporting questionnaires using VAS [21,22,23]. Our results had shown the same trend as previous studies. Thus, TENS treatment reduced masticatory muscle pain based on evidence that the release of analgesic endogenous opioids depends on the TENS frequency [32,33].

For maximum mouth opening without pain, there was significant interaction between the two groups. Furthermore, the TMD group had a significant increase in maximum mouth opening after TENS treatment as post-hoc test, and the control group had no significant increase after TENS. This result indicated that the tension along the membrane of the muscle fibers in TMD patients was relaxed by intervening TENS [23]. Three previous studies evaluating the mouth opening amplitude after TENS treatment showed the increased ratio after TENS were from 8.7% to 19.5 % [22,23,24]. The average of maximum mouth opening was 7.5% in this study, so that the value was slightly small.

For maximum bite force, since there was no significant interaction between TMD and control groups, they were the same trend before and after TENS treatment. Both TMD and control groups exhibited a significant increase in maximum bite force after TENS application compared to that before TENS application as univariate test. This indicates that TENS can quickly improve masticatory system functions in most subjects with TMD-related muscle pain and healthy control subjects. This result suggested that the TENS treatment might have activated trigeminal motoneuron. The previous study evaluated stomatognathic functional improvement by TENS treatment between active TENS group and placebo TENS group from the measurements of EMG activity during chewing after long duration treatment (50 min) for TMD patients [25]. This result showed that TENS had an efficacy to treat the masticatory muscle function in both groups; however, this did not include the control subjects to intervene TENS. Thus, this previous report did not sufficiently reveal that that TENS enhances motor function yet. The present study challenged whether the intervention of TENS had an efficacy to increase the masticatory muscle function in asymptomatic subjects such as control subjects as well. Other previous studies reported that TENS treatment improved muscle action but not reduce pain for healthy subjects with exercise-induced muscle pain [34]. Other than stomatognathic function area, stroke patients increased the trunk muscle activity by weight-shifting exercise with TENS in comparison with weight-shifting exercise with placebo TENS [35]. Therefore, TENS treatment might support the trunk muscle activity [36] and partially restore α-motoneuron activation [37]. However, past studies in stomatognathic area researched analgesic action in TMD and was not enough to investigate muscle activation for TENS treatment. Since our study focused on immediate activation of masticatory muscle, we did not investigate continuous trials.

As far as we are aware, this is the first attempt to objectively elucidate pain and discomfort relief by TENS using GRC scale. GRC scales are generally used in the musculoskeletal area and has ease of understanding and evaluating immediately in subjective global clinical changes before and after treatment for the participants and strong clinical relevance such as key strengths [30]. Although the TMD group showed significantly higher values of GRC scales than the control group, the power value was small. However, the pain intensity for TMD patients reduced significantly after the treatment, and maximum mouth opening and maximum bite force had increased.

Taken together with the present results, we suggested that TENS treatment immediately helped to reduce muscle pain and to increase muscle activity in stomatognathic area, however, clinicians should not only use physical therapy such as TENS but also motor therapy [35]. Further investigations are required to determine the optimal TENS protocol for maximal efficacy of alternate frequency TENS treatment in muscle pain related to TMDs, leading to guidelines for TENS application.

The present clinical trial of TENS treatment for TMD-related masticatory muscle pain is based on several assumptions limiting its generalizability. First, the subgroup of patients who received TENS treatment for masticatory muscle pain were moderate, weakening the conclusions regarding concurrent validity with GRC scales. Second, although control subjects were employed with almost the same age and gender difference as TMD patients, the background history in control subjects were unclear such as sleep bruxism, tooth contact habit, and social situations. As the control subjects were randomly employed, they might have various background history the same as TMD patients. Future study is needed to divide the category of the background history and to analyze more detailed statistical investigations. Third, this study did not include placebo TENS treatment group of TMD patients. In three previous reports, TMD patients were randomly divided into active TENS group and placebo TENS group and compared the treatment outcome between the two groups [25,38,39]. These reports focused on effect of TENS treatment for various situations such as changing frequency, electric power, and phase duration. However, this present study focused on the muscle activation using TENS intervention. Thus, by intervening to healthy control subjects without TMD symptoms, TENS was needed to verify activation for the motor function. Finally, seven dental clinicians who treated a lot of TMD patients for many years were involved in this clinical trial and used the TENS device to treat patients with TMD-related muscle pain. Since the present study is a clinical trial, in order to use the same treatment method to apply the patients, one instructor educated them how to use all TENS procedures. Since this study eliminated interindividual errors and differences in TENS treatment outcomes and to ensure standardization, they had been trained sufficiently before the start of the clinical trial a number of times. However, the intraclass correlation coefficients among the seven dental clinicians were not determined. Because of careful preparation to treat patients, the present results showed a normal dispersion of the data and the hypothesis that TENS quickly attenuates TMD-related muscle pain and activates masticatory function was not rejected. The authors consider that these results should be interpreted with caution for clinicians.

## 5. Conclusions

We conclude that TENS treatment might quickly relieve pain in masticatory muscles and improve masticatory system functions in patients with TMD-related muscle pain. On the other hand, masticatory motor function quickly improved in both the TMD and control subjects under the same conditions. TENS treatment could improve motor function as well as remove muscle pain in the stomatognathic area.

## Figures and Tables

**Table 1:** Comparison between temporomandibular disorders (TMD) patients and control subjects according to number of participants, age, and output Power of TENS device.

	TMD Patients	Controls	*p* Value
Number of Male	9	17	0.09 **
Number of Female	27	22	
Age *	56.8 ± 17.2	52.5 ± 17.6	0.82
Male	51.3 ± 25.3	55.23 ± 15.7	0.67
Female	58.6 ± 13.7	50.3 ± 19.0	0.09
Output Power	13.8 (11.0–17.0)	15.0 (12.0–17.0)	0.28

Mean ± SD: parametric distribution with student t-test; Median (IQR: first quartile – third quartile): non-parametric distribution with Mann-Whitney U test; *: total age of participants. **: Statistic analysis as Chi-Square test.
